# Recent Advances in Microwave and Ultrasound Assisted Synthesis of Quinoline Derivatives as Anticancer Agents

**DOI:** 10.1002/cbdv.71675

**Published:** 2026-09-20

**Authors:** Rohit Pal, Gurubasavaraja Swamy Purawarga Matada, Ghanshyam Teli, Manjushree B V, Vipin K. Pandey, Shourya Pratap

**Affiliations:** ^1^ Integrated Drug Discovery Centre, Department of Pharmaceutical Chemistry Acharya & BM Reddy College of Pharmacy Bengaluru Karnataka India; ^2^ School of Pharmacy Sangam University, Atoon Bhilwara Rajasthan India; ^3^ Arni School of Pharmacy Arni University Tanda Himachal Pradesh India

**Keywords:** anticancer, microwave‐assisted synthesis, quinoline, synthetic strategies, ultrasound‐assisted synthesis

## Abstract

Quinoline derivatives represent a privileged class of heterocyclic compounds with remarkable structural diversity and broad‐spectrum pharmacological activities, particularly in cancer therapy. Owing to their ability to modulate key oncogenic signaling pathways, quinoline‐based scaffolds have gained considerable attention in anticancer drug discovery. However, conventional synthetic routes for quinoline derivatives often suffer from prolonged reaction times, harsh conditions, low atom economy, and environmental concerns. In this context, green and sustainable synthetic methodologies have emerged as viable alternatives to address these limitations. Among them, microwave‐ and ultrasound‐assisted synthesis (UAS) have proven to be highly efficient non‐classical activation techniques, offering rapid reaction rates, improved yields, enhanced selectivity, and reduced energy consumption. This review provides a comprehensive overview of recent advances in the microwave‐ and UAS of quinoline derivatives with anticancer potential. Emphasis is placed on reaction efficiency, eco‐friendly reaction conditions, and the biological relevance of the synthesized compounds, including their cytotoxicity profiles, enzyme inhibition activities, and apoptosis‐inducing mechanisms. Collectively, this compilation highlights the integration of green chemistry principles into medicinal chemistry workflows and underscores the potential of sustainable synthetic strategies for the development of potent and selective quinoline‐based anticancer agents.

## Graphical abstract

Green microwave‐ and ultrasound‐assisted synthesis enables the rapid, sustainable preparation of quinoline‐based anticancer agents with improved reaction efficiency, higher yields, and enhanced biological activity.

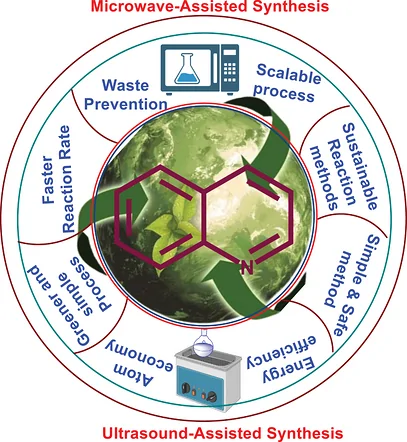

**Abbreviations**
A549: human lung adenocarcinoma cell line
Akt: protein kinase B
ATP: adenosine triphosphate
BCR‐ABL: breakpoint cluster region–Abelson tyrosine kinase
CDKs: cyclin‐dependent kinases
CuAAC: copper(I)‐catalyzed azide–alkyne cycloaddition
DBU: 1,8‐diazabicyclo[5.4.0]undec‐7‐ene
DNA‐PK: DNA‐dependent protein kinase
DPPH: 2,2‐diphenyl‐1‐picrylhydrazyl
EGFR: epidermal growth factor receptor
FDA: Food and Drug Administration
FGFR: fibroblast growth factor receptor
GI_50_
: growth inhibitory concentration (50%)
HBL‐100: human breast epithelial cell line
HDAC: histone deacetylase
HER‐2: human epidermal growth factor receptor 2
IC_50_
: half‐maximal inhibitory concentration
MAS: microwave‐assisted synthesis
MCF‐7: Michigan Cancer Foundation‐7 Breast Cancer Cell Line
MET: mesenchymal–epithelial transition factor
MTT: 3‐(4,5‐dimethylthiazol‐2‐yl)‐2,5‐diphenyltetrazolium bromide
NSCLC: non‐small cell lung cancer
PBS: phosphate‐buffered saline
PDGFR: platelet‐derived growth factor receptor
PI3K: phosphoinositide 3‐kinase
ROS: reactive oxygen species
SAR: structure–activity relationship
THF: tetrahydrofuran
TKI: tyrosine kinase inhibitor
UAS: ultrasound‐assisted synthesis
VEGF: vascular endothelial growth factor
VEGFR: vascular endothelial growth factor receptor

## 1 Introduction

Organic and medicinal chemistry have played a vital role in the discovery and development of structural diverse heterocyclic scaffolds with wide spectrum pharmacological significance. Among them, quinoline derivatives represent an important class and can be distinguished by their structural diversity and broad applicability [1, 2, 3, 4, 5]. Naturally occurring and synthetic quinolines have found extensive applications in pharmaceutical and industrial domains, particularly due to their pronounced biological activities [6, 7]. Consequently, quinoline scaffolds have been widely explored in synthetic organic, agrochemicals, dyes, and medicinal chemistry, leading to the development of numerous synthetic approaches aimed at improving efficiency and sustainability [7, 8, 9, 10].

**FIGURE 1:**
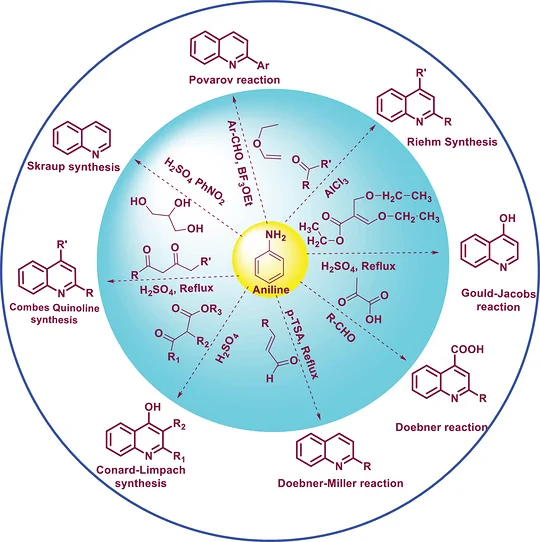
Schematic representation of classical synthetic routes for quinoline derivatives originating from aniline, illustrating key named reactions, including Skraup, Doebner–Miller, Povarov, Combes, Conrad–Limpach, Gould–Jacobs, Doebner, and Riehm syntheses, along with typical reagents and reaction conditions [21].

Quinoline is a privileged scaffold that contains nitrogen and is commonly termed as benzopyridine or benzazine. It was first isolated from crude coal tar by Friedlieb Ferdinand Runge in 1834 and emerged as a vital congener for industrial and medicinal chemistry due to its diverse bioactive properties and wide synthetic flexibility [11, 12, 13, 14, 15, 16, 17, 18, 19]. Several synthetic pathways have been developed for the synthesis of quinoline, including catalyst‐free synthesis, Brønsted acid/base catalysts, and transition metal catalysts. Among the most widely employed classical synthetic routes are the Friedländer, Pfitzinger, Povarov, Skraup, Doebner, and Conrad–Limpach–Knorr syntheses (Figure 1) [12, 20, 21]. The incorporation of nitrogen in quinoline depends on specific reaction conditions and the desired quinoline derivative [21]. Although aniline most commonly serves as the starting material, some synthetic procedures utilize ammonia, hydrazine, nitriles, and nitroso compounds as starting materials.

Nevertheless, the majority of traditional methods necessitate severe reaction conditions, high temperatures, potent acid catalysts, and hazardous solvents. These restrictions frequently lead to decreased sustainability and poor selectivity [22, 23]. As a result, several modifications to these reactions utilizing organo and transition metal‐catalyzed synthesis, photocatalytic, electrocatalytic, microwave, and ultrasound‐mediated synthesis have been documented more recently [24, 25, 26].

With the idea of “meeting the needs of the present generation without compromising the needs of the future generation,” sustainability has drawn a lot of attention in recent decades from both industry and society. In the progress of sustainable development, the principle of green chemistry addresses the fundamental scientific challenges of protecting both the environment and human health [27, 28, 29]. As a result, contemporary green chemistry approaches are becoming more and more well‐liked as unconventional means of achieving quick organic changes [30]. The use of predictable hazardous volatile organic solvents and the production of hazardous compounds could be reduced or eliminated by non‐classical methods that embrace green chemistry principles. Among the various non‐classical approaches, microwave and ultrasound‐assisted synthesis (UAS) are the most promising for the rapid synthesis of various organic entities [31, 32, 33, 34].

**FIGURE 2:**
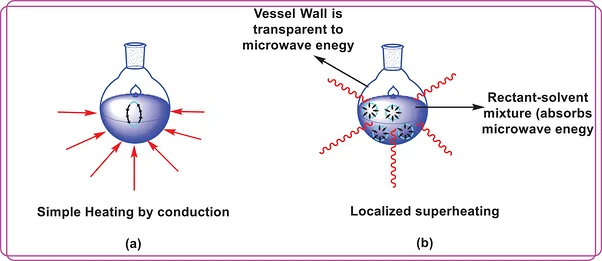
Schematic comparison of (a) conventional conductive heating, where heat is transferred from the vessel wall to the reaction mixture, and (b) microwave‐assisted heating, illustrating direct interaction of microwave energy with polar molecules and ions, leading to rapid and localized superheating [45].

The classical method for conducting chemical synthesis involves the use of an external source of heating, such as a hot plate, heating mantel, water bath, sand bath, or oil bath. Prior to reaching the solvent and reactants, heat is first transmitted via the reaction vessel's walls in these conventional methods (Figure 2a) [35, 36]. Because this heating method depends on the thermal conductivity of several components that need to be stabilized, energy transmission is very slow and ineffective [32, 37, 38]. Additionally, the temperature of the reactor/reaction vessel was higher than the reaction mixture until adequate time had elapsed to allow the reaction vessel and contents inside the vessel to establish thermal equilibrium, which took more time [34, 39]. Moreover, it usually takes more time to reach thermal equilibrium since the temperature of the reaction vessel is higher than the temperature of the reaction mixture [34, 38, 40]. Additionally, conductive heating restricts accurate process control and frequently calls for manual intervention. The method is labor‐intensive since cooling mechanisms must be implemented to lower the system's internal temperature after the reaction is finished, and the external heat source must be removed. In contrast, microwave heating represents a fundamentally different technique. As illustrated in Figure 2b, microwaves interact directly with molecules present in the reaction mixture, resulting in rapid and uniform temperature elevation [41]. This mechanism does not depend on the thermal conductivity of the reaction vessel. Instead, it enables instantaneous and localized heating of materials capable of responding through ionic conduction or dipolar polarization (dipole rotation) [39, 40, 41, 42, 43, 44].

Microwave heating offers several advantages consistent with green chemistry principles. It significantly reduces reaction times (often to minutes or seconds) and allows remote energy transfer without direct contact between the heat source and reactants. Energy input can be immediately stopped by turning off the power, and the system exhibits lower thermal inertia than conventional conductive heating [46, 47, 48]. Unlike traditional surface heating, microwave energy is distributed throughout the reaction mass, enabling rapid and uniform heating, especially when components strongly absorb microwave radiation. The technique is suitable for parallel or sequential synthesis and often provides cleaner reactions, milder conditions, simpler workup, improved selectivity (including regio‐ and stereoselectivity), potential catalyst‐free conditions, and the use of ecofriendly solvents [45, 46, 47, 48].

**FIGURE 3:**
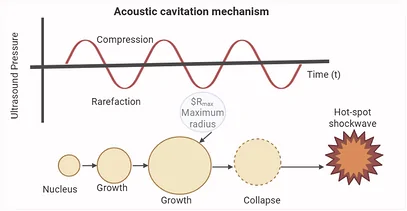
Acoustic cavitation mechanism in UAS showing the cyclic process of bubble nucleation, growth under rarefaction, expansion to a maximum radius (R_max), and subsequent collapse during compression, producing localized high‐temperature and high‐pressure hot spots that enhance reaction kinetics.

In a manner analogous to microwave irradiation, UAS represents another powerful non‐classical activation technique that has gained considerable attention in green and sustainable chemistry [49]. Unlike conventional conductive heating, ultrasound irradiation introduces energy into the reaction system through acoustic cavitation, which involves the formation, growth, and violent collapse of microscopic bubbles in a liquid medium (Figure 3) [49, 50]. The implosive collapse of these cavitation bubbles generates localized hot spots with transient temperatures of several thousand Kelvin and pressures exceeding hundreds of atmospheres, while the bulk reaction medium remains at near‐ambient conditions. This unique phenomenon enables chemical transformations to proceed under milder and more controlled conditions than traditional thermal methods. The energy transfer in ultrasound‐assisted reactions occurs directly within the reaction medium and is independent of the thermal conductivity of the reaction vessel, thereby overcoming many limitations associated with conductive heating [51, 52]. The localized high‐energy microenvironments produced during cavitation enhance mass transfer, molecular collisions, and reaction kinetics, resulting in accelerated reaction rates and improved product yields. Furthermore, ultrasound irradiation promotes efficient mixing and surface activation, particularly in heterogeneous systems, facilitating reactions that are otherwise sluggish or inefficient under conventional conditions.

**FIGURE 4:**
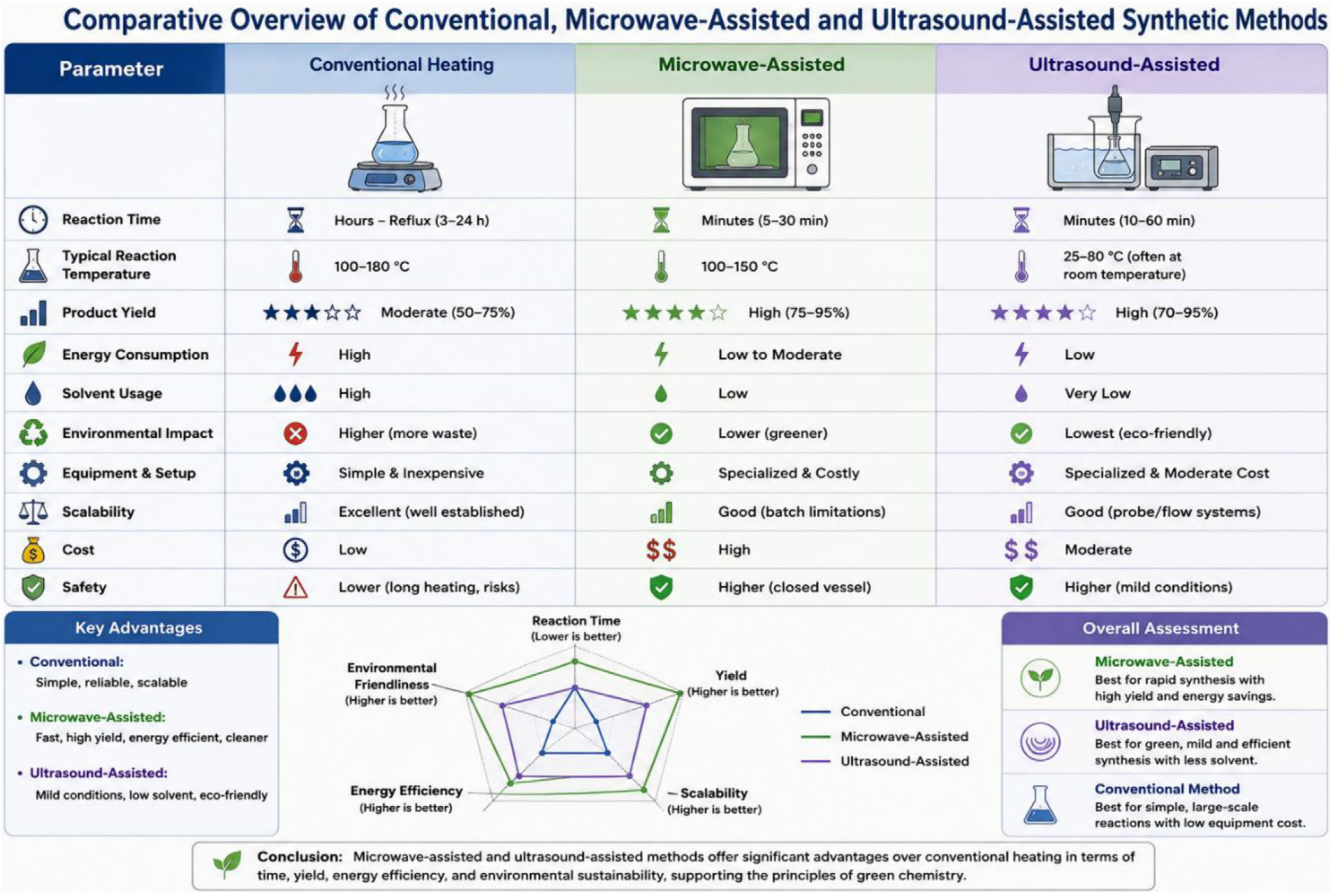
Schematic comparison of conventional, microwave‐assisted, and ultrasound‐assisted synthetic methodologies for quinoline derivatives. The figure summarizes the relative advantages and limitations of each approach in terms of reaction time, product yield, energy efficiency, solvent consumption, environmental sustainability, scalability, operational safety, and industrial applicability, highlighting the benefits of modern green synthetic technologies. The figure was created with the assistance of OpenAI ChatGPT (GPT‐5.6 Luna; accessed July 15, 2026). The authors directed the figure development and reviewed, edited, and approved the final illustration to ensure scientific accuracy and consistency with the underlying concepts.

From a green chemistry perspective, UAS offers several notable advantages. It enables shorter reaction times, reduced energy consumption, and lower reaction temperatures, while often eliminating the need for harsh reagents or excess catalysts. Many ultrasound‐mediated reactions can be conducted under solvent‐free or aqueous conditions, thereby minimizing environmental impact. Additionally, ultrasound irradiation has been shown to improve regio‐ and chemoselectivity, suppress side reactions, and allow easier product isolation and purification. Owing to these attributes, microwave and ultrasound‐assisted methodologies have been widely employed in the synthesis of diverse heterocyclic frameworks, including biologically active quinoline derivatives, aligning well with the principles of sustainable and environmentally benign chemical synthesis. The illustrative compression of conventional, microwave, and UAS is given in Figure 4.

### 1.1 Quinoline as a Privileged Scaffolds as in Medicinal Chemistry

**FIGURE 5:**
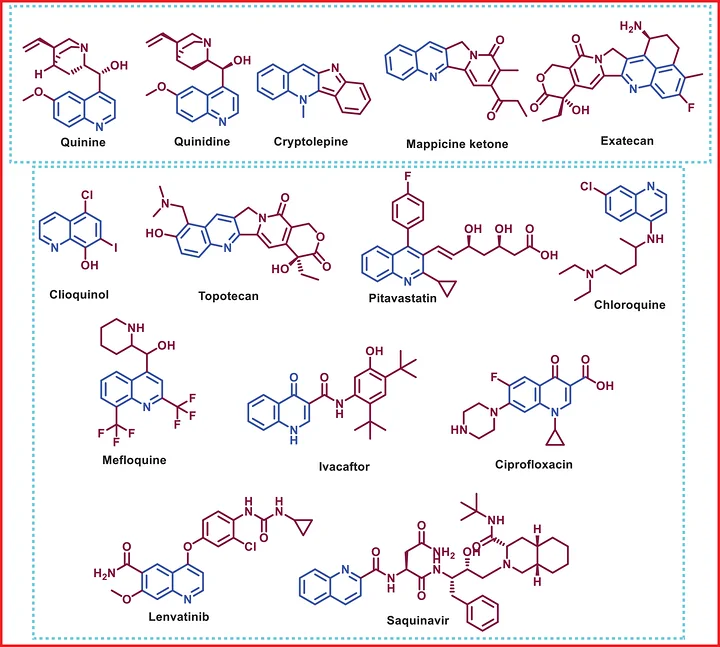
FDA‐approved quinoline derivatives with different pharmacological activities.

There are numerous uses for quinoline and related quinoline derivatives in the manufacturing of paints, dyes, pesticides, and antibiotic compounds [53]. But the quinoline structure also serves as a basic framework for many natural products and medicinal chemistry for the synthesis of various essential drugs [11, 54]. A quinoline ring is typically found in numerous synthetic antimalarial [55, 56, 57], antibacterial [58], antifungal [59], and anti‐inflammatory drugs [15], carbonic anhydrase inhibitory [60], antioxidant [61], and natural substances with potent properties in inhibiting the growth of parasites, such as quinine, chloroquine [62], and amodiaquine, have been employed in the treatment of malaria (Figure 5). Some quinoline compounds have shown antibacterial qualities, especially against the tuberculosis‐causing *Mycobacterium tuberculosis*. Quinolines have demonstrated antifungal properties against a variety of fungal infections. For example, derivatives of styrylquinoline are efficient against Candida albicans, a common fungal pathogen that causes a variety of diseases [63].

Pitavastatin and other quinoline‐based drugs are used to lower cholesterol and to treat cardiovascular conditions [64]. Because quinoline derivatives can interact with neurotransmitter systems and receptors, they have been studied for their potential in treating neurodegenerative diseases like Parkinson's and Alzheimer's [65, 66]. Certain quinoline compounds have anti‐inflammatory qualities and influence immunological responses, indicating possible uses in inflammatory and autoimmune diseases [15]. Quinoline compounds are excellent prospects for drug development in a variety of therapeutic areas, including neurological disorders, cancer, and infectious diseases, due to their varied pharmacological actions [12].

### 1.2 Role of Quinoline Scaffold in Anticancer Drugs

**FIGURE 6:**
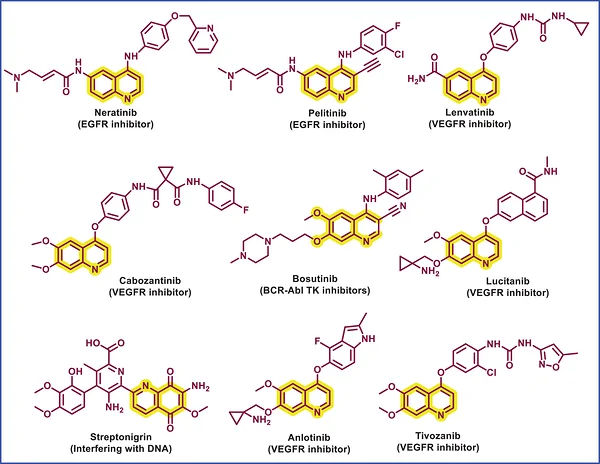
Representative quinoline‐based anticancer agents targeting key molecular target involve in cancer. The figure highlights clinically relevant quinoline derivatives acting as EGFR inhibitors (neratinib and pelitinib), VEGFR inhibitors (lenvatinib, cabozantinib, lucitanib, anlotinib, and tivozanib), BCR‐ABL tyrosine kinase inhibitors (bosutinib), and DNA‐interfering agents (streptonigrin), demonstrating the structural versatility of the quinoline scaffold in cancer therapy.

In the field of oncology, quinoline has great importance in terms of pharmacological action due to its ability to modulate key oncogenic signaling pathways (Figure 6) [67], while permitting widespread structural alterations to improve potency, selectivity, and pharmacokinetic properties. Quinoline‐based anticancer agents, such as lenvatinib and other kinase inhibitors, exert their therapeutic effects by targeting critical pathways involved in tumor growth, angiogenesis, and disease progression [68]. These approved drugs exemplify the clinical success and translational potential of the quinoline scaffold. Complementing this, the clinical relevance of quinoline derivatives is further supported by the ongoing and completed clinical trials summarized in Table 1, which include investigational and repurposed quinoline‐containing compounds evaluated across multiple cancer indications, such as non‐small cell lung cancer, cervical intraepithelial neoplasia, multiple myeloma, and refractory solid tumors. The interventions target multiple oncogenic mechanisms, including autophagy modulation (hydroxychloroquine), immune response activation (imiquimod) [69], and kinase inhibition (SNS‐595 and silmitasertib/CX‐4945) [70], highlighting the therapeutic versatility and clinical relevance of quinoline‐based scaffolds in contemporary oncology research.

**TABLE 1:** Clinical‐stage evaluation of quinoline‐based and quinoline‐related anticancer agents, highlighting investigational drugs undergoing Phase I–II clinical trials across multiple cancer indications.

Study title	Clinical Trials.gov Identifier	Intervention	Status	Sponsor	Structure
Hydroxychloroquine combined with gemcitabine in the treatment of advanced non‐small cell lung cancer [72]	NCT05647330	Hydroxychloroquine combined with gemcitabine	Phase 2	Henan Cancer Hospital	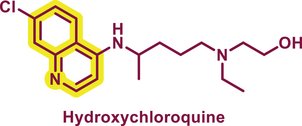 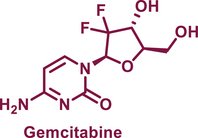
Predicting response In cervical intraepithelial neoplasia to topical imiquimod treatment (PRedICT‐TOPIC) [73]	NCT05405270	Imiquimod	Observational (Patient Registry)	Catharina Ziekenhuis Eindhoven	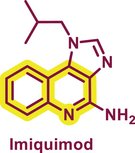
Safety and efficacy clinical study of SNS‐595 for second‐Line therapy in patients with advanced NSCLC [74]	NCT00252382	SNS‐595 Injection	Phase 2	Sunesis Pharmaceuticals	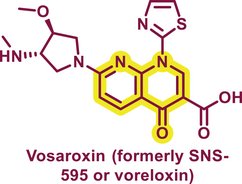
Silmitasertib (CX‐4945) in combination with chemotherapy for relapsed refractory solid tumors [75]	NCT06541262	Silmitasertib, irinotecan, temozolomide, and vincristine	Phase 1/2	Milton S. Hershey Medical Center	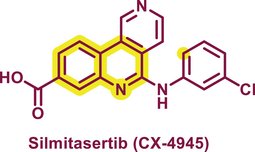 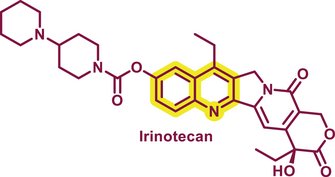 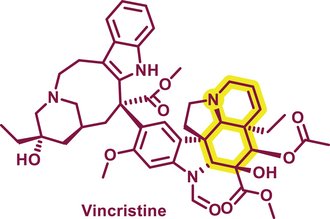 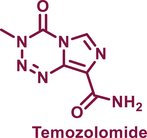
Study of CX‐4945 in patients with relapsed or refractory multiple myeloma [76]	NCT01199718	CX‐4945	Phase 1	Cylene Pharmaceuticals	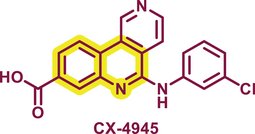

The integration of quinoline derivatives towards anticancer properties is arbitrated through miscellaneous mechanisms, including inhibition of receptor tyrosine kinases (e.g., EGFR and VEGFR), suppression of the PI3K/Akt/mTOR signaling pathway, inhibition of topoisomerases I and II, disruption of tubulin polymerization, modulation of cyclin‐dependent kinases (CDKs), and epigenetic regulation through histone deacetylase (HDAC) inhibition. In addition to targeting these signaling pathways, quinoline derivatives have been reported to induce apoptosis, arrest cell‐cycle progression, inhibit metastasis and angiogenesis, encourage reactive oxygen species (ROS)‐mediated oxidative stress, and dazed multidrug resistance in different types of cancer. These complex mechanisms emphasize the beneficial potential of the quinoline scaffold in the design and development of next‐generation anticancer agents.

Despite these developments, and advancement of synthetic procedure, the conventional methodologies of quinoline derivatives often depend on extended reaction times, elevated temperatures, risky reagents, toxic organic solvents, and multistep synthetic procedures that produced noteworthy amounts of chemical waste. These approaches are frequently associated with underprivileged atom economy, high energy utilization, labor‐intensive purification methods, and inadequate overall process competence, making them less suitable for sustainable and large‐scale production. Moreover, the utilization of environmentally destructive solvents and reagents increases apprehensions regarding occupational safety, environmental pollution, and regulatory compliance. Collectively, these restrictions emphasize the urgent requirement for greener, more well‐organized, and economically viable synthetic procedures that minimize waste generation, reduce energy requirements, improve reaction competence, and align with the principles of green chemistry while simplifying the rapid expansion of quinoline‐based anticancer agents.

The growing demand for sustainable synthetic strategies and the promising anticancer potential of quinoline derivatives have prompted extensive research into the design, green synthesis, and pharmacological evaluation of novel quinoline‐based compounds. Recently, several high‐quality literatures published highlight quinoline architectures and environmentally benign strategies. For instance, in 2024 compilations by Kumaraswamy and team [21] and Babita et al. [30], comprehensive trends in current green pathways have been outlined, while Parmar and Patel, in the year 2025 [71], reported an extensive comparison between classical and green one‐pot protocols for pharmacological active quinoline derivatives. However, a critical survey of the reported literature reveals clear thematic and structural gaps that our review exceptionally structured to address. First, there is gap for chemical–biological connect in typical literature. Most of the existing synthetic literature primarily emphasizes augmenting green reaction parameters (such as catalyst reusability, atom economy, or solvent‐free conditions) but limits biological tracking to nonspecific baseline screenings.

Current review explicitly bridges this gap by unswervingly connecting green chemical productivity such as the high 80%–95% yields obtained during multicomponent spiro‐quinoline condensations with extremely specific oncological pathways, including multi‐receptor tyrosine kinase inhibition (EGFR, VEGFR, HER‐2), topoisomerase I/II suppression, and precise quantitative apoptotic ratios. Moreover, the review presents a highly specialized, scaffold‐centric classification structure–activity relationship (SAR) according to their exact substitution positions (C‐4, C‐7, C‐8, and ortho‐fused frameworks) on the core quinoline nucleus. It further sheds light on recent clinical developments by highlighting quinoline‐based anticancer agents in clinical trials (e.g., Silmitasertib and SNS‐595) and discusses the future potential of merging microwave‐ and UAS with artificial intelligence (AI) and machine learning (ML) for predictive retrosynthesis, reaction optimization, and accelerated drug discovery. Therefore, this review serves as an applied guide for the rational design and green synthesis of next‐generation quinoline‐based anticancer agents.

## 2 Current Landscape of Microwave‐ and Ultrasound‐Assisted Methodologies for the Synthesis of Quinoline Derivatives as Potential Anticancer Agents

### 2.1 Synthesis of Quinoline Derivatives as Potential Anticancer Agents by Means of Microwave‐Assisted Synthesis (MAS)

**SCHEME 1:**
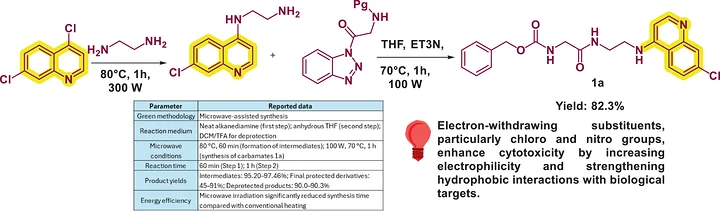
Microwave‐assisted synthesis of 7**‑**chloroquinoline derivative (**1a**).

In contemporary organic and medicinal chemistry, MAS has emerged as a very effective and environmentally friendly technique [77, 78, 79]. In contrast to traditional heating techniques, microwave irradiation enables rapid, uniform heating by direct coupling with polar solvents or reactants, drastically reducing reaction times and energy consumption. Because of these benefits, MAS is now widely recognized as a useful instrument for drug research and discovery [78, 80]. Utilizing these properties, in the year 2026, Ezugwu and co‐workers designed, synthesized, and evaluated 7**‑**chloroquinoline as a potential cytotoxic agent. The synthesized compounds were obtained through a benzotriazole‐mediated approach via microwave synthesis. The in vitro anticancer potential of a novel family of 7‐chloroquinolinyl benzyl amino carbamate derivatives (Scheme 1) was assessed using the conventional MTT assay, and the cytotoxic activity was evaluated against LNCaP, A2780, and MCF‐7. Following a 24‐h treatment period, cell viability was assessed, and antiproliferative potency was ascertained by calculating IC_50_ values. Compound **1a** was shown to be the most active analogue among the produced derivatives, demonstrating notable cytotoxicity in all three cancer cell lines with IC_50_ values of 6.61 µg/mL (LNCaP), 2.81 µg/mL (A2780), and 5.69 µg/mL (MCF‐7). However, compound **1a** was less potent than docetaxel against all tested cancer cell lines, with IC_50_ values ranging from 0.37 to 0.58 µg/mL [81].

**SCHEME 2:**
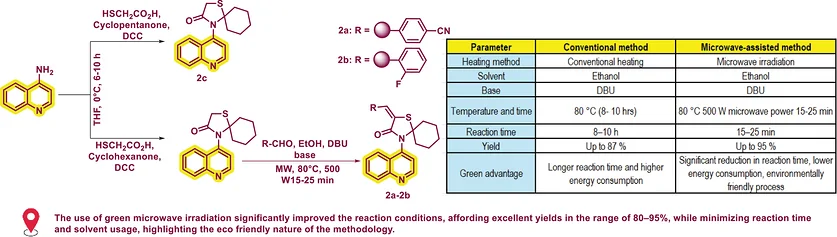
Microwave‐assisted three‐component condensation synthesis of quinoline‐substituted aromatic aldehydes.

In 2025, microwave‐assisted three‐component condensation of quinoline‐substituted aromatic aldehydes (Scheme 2) utilizing DBU in ethanol was reported by Humal and colleagues. The synthesis has advantages such as reduced reaction time, solvent usage, and good yields (80%–95%). Among the reported series, compounds **2a**, **2b**, and **2c** demonstrated significant cytotoxic activity against MCF cells with IC_50_ values of 9.44, 15.39, and 1.611 µM, respectively. However, all the compounds were inferior to standard (doxorubicin, IC_50_ = 1.20 µM). Among all the derivatives, Compound **2c** exhibited activity comparable to doxorubicin, with an IC_50_ value only 1.34‐fold higher than that of the reference drug [82].

**SCHEME 3:**
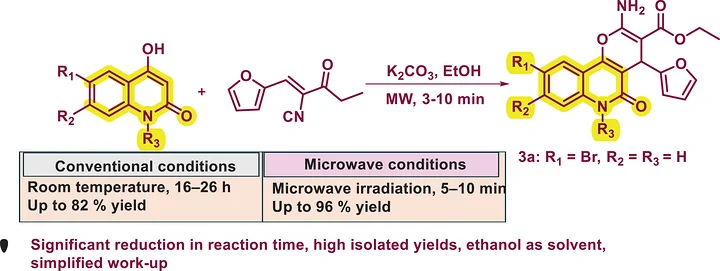
Microwave‐assisted synthesis of pyrano‐quinolone derivatives.

In the same year, Aly and colleagues described a synthetic method using microwaves to create new pyrano‐quinolone derivatives (Scheme 3) that have the ability to induce apoptosis for the treatment of lung cancer. With an IC_50_ value of 35.10 µM, derivative **3a** demonstrated the strongest cytotoxic activity among the produced compounds against the A549 lung cancer cell line as compared to the levofloxacin (IC_50_: 410.10 µM). Compound **3a** also showed significant inhibitory activity against topoisomerase II, with an IC_50_ value of 45.19 µM, albeit somewhat less efficacy than the reference medication etoposide (IC_50_ = 34.99 µM). Apoptosis analysis revealed that compound **3a** markedly enhanced apoptotic cell death in lung cancer cells (38.49‐fold increase), with total apoptosis reaching 20.4% (12.29% early and 8.11% late), compared to 0.53% in untreated controls. In contrast, necrosis was only modestly elevated (3.73% vs. 1.66%; 2.24‐fold), indicating that compound **3a** primarily induces apoptosis rather than necrosis in lung cancer cells [83].

**SCHEME 4:**
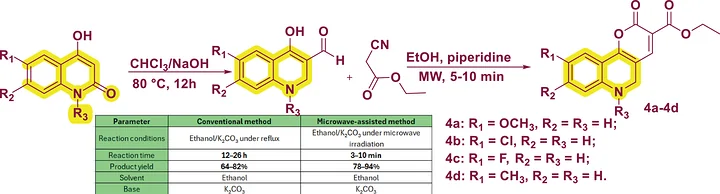
Microwave‐assisted synthesis of pyrano[3,2‐*c*]quinoline‐3‐carboxylate derivatives (**4a–4d**).

In 2024, the same group synthesized Pyrano[3,2‐*c*]quinoline‐3‐carboxylate derivatives (Scheme 4) via a piperidine‐catalyzed condensation of 4‐hydroxy‐2‐oxo‐1,2‐dihydroquinoline‐3‐carbaldehydes with ethyl cyanoacetate in ethanol. The anticancer potential of the synthesized derivatives was evaluated against a panel of cancer cell lines, including Panc‐1, MCF‐7, HT‐29, and A549. The reference drug doxorubicin exhibited promising anticancer activity (GI_50_ = 1.10 µM), while the synthesized compounds **4a–4d** showed comparable potency, with GI_50_ values ranging from 1.30 to 5.90 µM. Notably, compound **4a** emerged as the most potent derivative, displaying significant EGFR inhibitory activity and being only 1.3‐fold less active than erlotinib (IC_50_ = 80 nM) [84].

**SCHEME 5:**
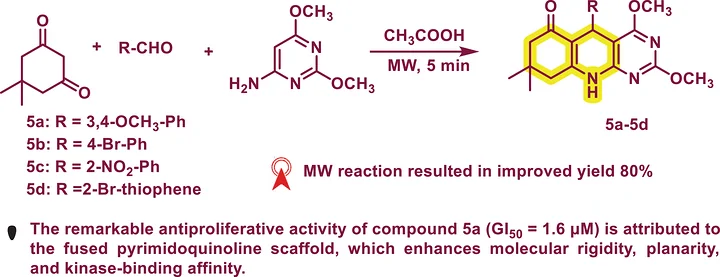
Microwave‐assisted synthesis of pyrimido‐quinoline hybrids (**5a–5d**).

Patel and collaborators prepared a novel series of pyrimido‐quinoline hybrids (**5a–5d**) (Scheme 5) through a microwave‐assisted multicomponent technique as potential anticancer agents. Among the series of tested derivatives, compound **5a** confirmed significant activity without GI_50_ = 1.6 µM against the HBL‐100 cancer cell line. Furthermore, compounds **5a–5d** showed notable antitumor activity across the panel of cancer cell lines, with GI_50_ values varying from 1.6 to 19 µM, comparable range to cisplatin (GI_50_: 1.8–23 µM) [85].

**SCHEME 6:**
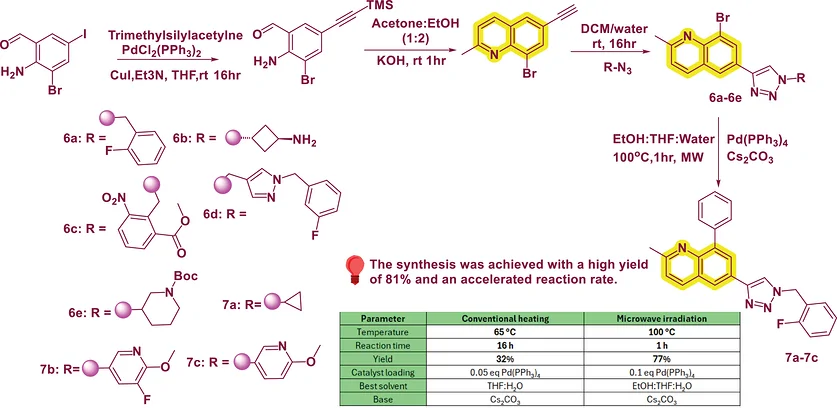
Microwave‐assisted synthesis of quinoline‐triazole derivatives through classical Suzuki coupling (**6a–6e** and **7a–7c**).

Utilizing MAS through classical Suzuki coupling, Venkata et al. synthesized two different series of quinoline‐triazole hybrids **(6a–4e** and **7a–7c)** (Scheme 6) and tested them as potential antiproliferative agents. The series containing 5‐bromo‐1H‐1,2,3‐triazol‐4‐yl‐2‐methylquinoline (**6a–6e**) derivatives was developed. In the first step, the bromo‐substituted aniline undergoes Sonogashira coupling with trimethylsilylacetylene in the presence of PdCl_2_(PPh_3_)_2_/CuI and Et_3_N in THF to afford the TMS‐protected intermediate. Further, the TMS group is removed using KOH in EtOH to generate the terminal alkyne, which subsequently undergoes Cu‐catalyzed azide–alkyne cycloaddition (CuAAC) to form the quinoline–triazole derivatives. Another series (**7a–7c**) was prepared via a derivative (5a–5e) via a microwave‐assisted Suzuki coupling reaction catalyzed by copper azide‐alkyne cycloaddition (CuAAC). The synthesized derivatives were assessed for their anticancer efficacy against the MDA‐MB‐231 and B16F10 cell lines. Compounds **6a**, **6b**, and **6e** displayed pronounced cytotoxic effects against the B16F10 cell line, with IC_50_ values of 16.395, 13.446, and 18.88 µM, respectively. Meanwhile, compounds **6c**, **6d**, **7a**, and **7b** showed appreciable inhibitory activity toward the MDA‐MB‐231 cell line, exhibiting IC_50_ values of 17.063, 24.156, 20.699, and 19.676 µM, respectively. Compounds **6a** and **6b** were approximately 2.57‐fold and 2.11‐fold more potent than the standard (BG‐45) with IC_50_ value of 34.586 µM, respectively [86].

**SCHEME 7:**
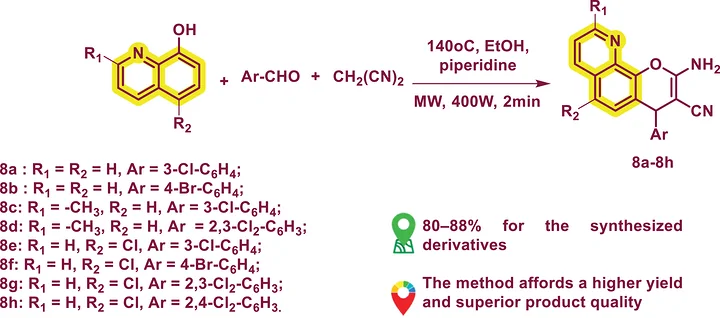
Microwave‐assisted synthesis of pyrano[3,2‐*h*] quinoline‐3‐carbonitrile scaffold (**8a–8h**).

By employing the microwave techniques, Fouda and co‐researchers produced pyrano‐quinolines congeners. The pyrano[3,2‐*h*] quinoline‐3‐carbonitrile scaffold (**8a–8h**) (Scheme 7) was synthesized by using the condensation of substituted aryl/hetaryl aldehydes with hydroxyquinoline analogues using one‐pot synthesis. The acquired series' cytotoxicity was examined against the cancer cell lines A549, HepG‐2, HCT‐116, and MCF‐7. Across the series of derivatives compounds, **8a** (IC_50_ = 1.42, 1.35, 1.87, and 1.23 µM), **8b** (1.17, 1.3, 1.41, and 38 µM), **8e** (0.9, 3.04, 0.7, and 2.67 µM), **8f** (0.99, 5.81, 1.23, and 3.1 µM), **8g** (2.47, 7.94, 1.48, and 15.6 µM), and **8h** (1.18, 3.01, 0.87, and 1.79 µM) showed significant anticancer potency among the tested cell lines (MCF‐7, HCT‐116, HepG‐2, and A549, respectively). Compounds **8c** (IC_50_ = 5.79 and 3.01 µM) and **8d** (IC_50_ = 1.24 and 1.83 µM) exhibited selective inhibition only in the MCF‐7 and HepG‐2 cell lines. Whereas, vinblastine (IC50 = 3.78 ± 0.01 µg/mL) less active. Flow cytometry was also used to assess the apoptosis and cell cycle arrest. Results indicated that compound **8a** demonstrated cell cycle arrest at G2/M phase in the A549 cell line and late apoptosis against MCF‐7, HepG‐2, and A549 cell lines [87].

**SCHEME 8:**
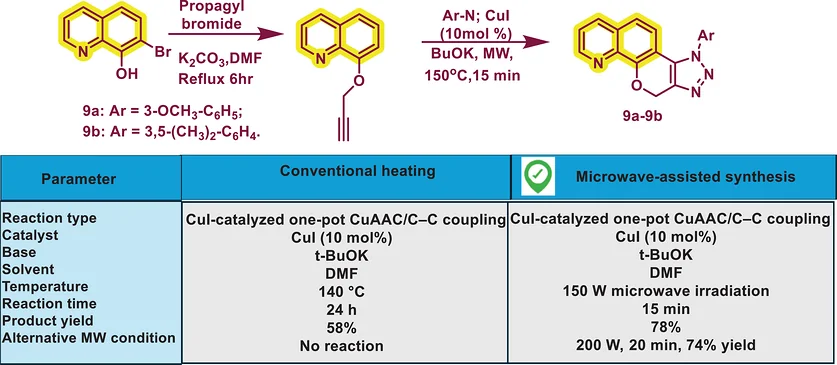
Microwave‐assisted synthesis of fused[1,2,3]triazolo‐pyrano[3,4‐*h*] quinolines derivatives (**9a–9b**).

In 2019, using a microwave reactor, Babu H. and his co‐authors created fused quinoline compounds and assessed their anticancer properties. Fused [1,2,3] triazolo‐pyrano[3,4‐*h*] quinolines (**9a–9b)** were produced and tested against a set of cancer cell lines (Scheme 8). Compound **9b** had considerable activity against MCF‐7, A‐549, and HeLa cell lines (IC_50_ = 16.88, 32.47, and 11.42 µM), whereas compound **9a** showed good activity against MCF‐7 and HeLa cell lines (IC_50_ = 31.44, 15.67 µM). These results are comparable to those of the standard drug doxorubicin against MCF‐7, A‐549, and HeLa cell lines (IC_50_ = 3.01, 1.08, and 1.35 µM) [88].

**SCHEME 9:**
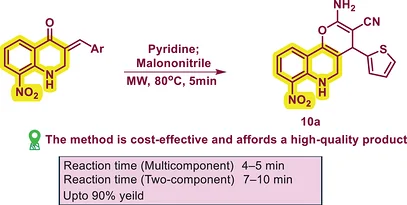
Microwave‐assisted multicomponent one‐pot approach synthesis of 8‐nitroquinoline (**10a**).

Using a multicomponent one‐pot approach, Arasakumar et al. developed an environmentally friendly microwave synthesis of 8‐nitroquinoline (Scheme 9) and assessed the compound's anticancer potential. The developed series was tested against MCF‐7 and A549 cell lines for potential anticancer activity. Compound **10a** was the most significant of all the derivatives, with IC_50_ values of 18.56 and 15.12 µM against the A549 and MCF‐7 cell lines, respectively, as compared with the reference (doxorubicin) drug (IC_50_: 15.12 and 18.56 µM) [89].

**SCHEME 10:**
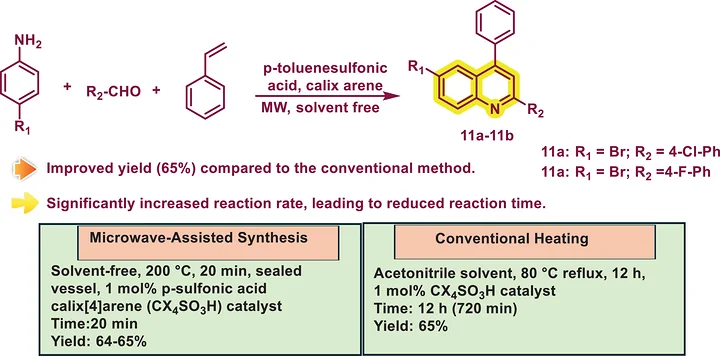
Microwave‐assisted synthesis of quinoline derivatives (**11a–11b**).

In the year 2017, Liberto and colleagues described the effective synthesis of quinolines, and the antineoplastic potential of the produced derivatives (**11a–11b**) (Scheme 10) was examined. Using p‐sulfonic acid calixarene (CX_4_SO_3_H) and microwave assistance, several quinolines were synthesized. The developed series was examined on Hep‐2C and NCI‐H226 cell lines. Among the different derivatives, compound **11a** had exceptional anticancer activity against the NCI‐H226 cell line (IC_50_ = 1.58 µM), whereas **11b** was highly effective against the Hep‐2C cell line (IC_50_ = 5 µM) [8].

**SCHEME 11:**
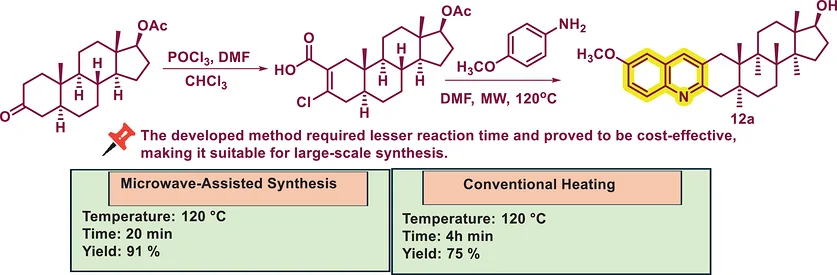
Microwave‐assisted synthesis of steroid‐quinoline hybrids (**12a**).

The series of steroid‐quinoline hybrids (**12a**) (Scheme 11) was synthesized by MAS and examined for their potential anticancer properties. Among the series of derivatives, compound **12a** showed strong activity against the cell lines C33A (IC_50_ = 10.2 µM), MCF‐7 (IC_50_ = 19.1 µM), and T47D (IC_50_ = 10.2 µM). Additionally, compound **12a** was examined for its ability to induce apoptosis and cell cycle arrest. According to the study, the G0/G1 phase's cell count considerably increased in comparison to the S and G2/M phases, and the caspase‐3 enzyme's activity dramatically enhanced as well [90].

**SCHEME 12:**
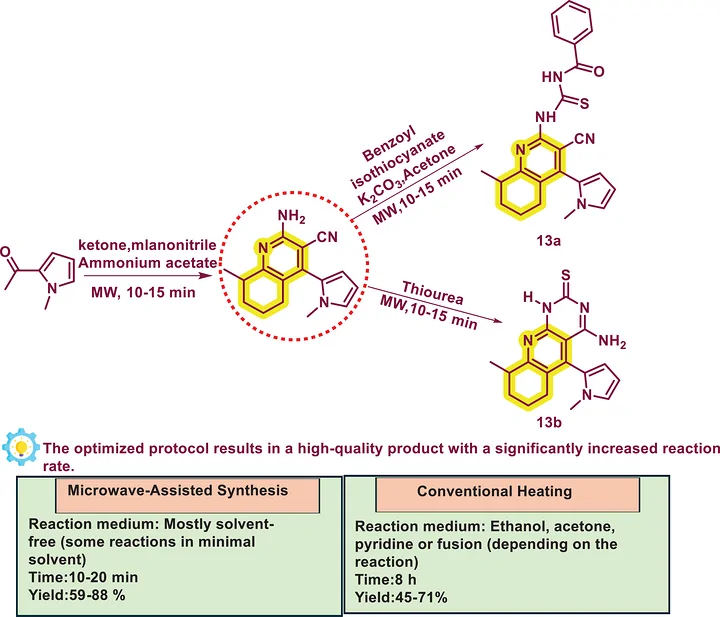
Microwave‐assisted synthesis of quinoline consolidated pyrimidine derivatives (**13a–13b**).

Rostom and associates used green synthesis to produce quinoline consolidated pyrimidine derivatives (**13a–13b**) (Scheme 12) as potential anticancer agents. The sets of derivatives were assessed using a panel of cancer cell lines through in vitro evaluation. The results of an in vitro analysis showed that compound **13b** had robust activity against the LOX IMVI cancer cell line (IC_50_: 10.7 µM), whereas compound **13a** had significant activity against the EKVX cancer cell line (IC_50_: 14.2 µM) [91].

**SCHEME 13:**
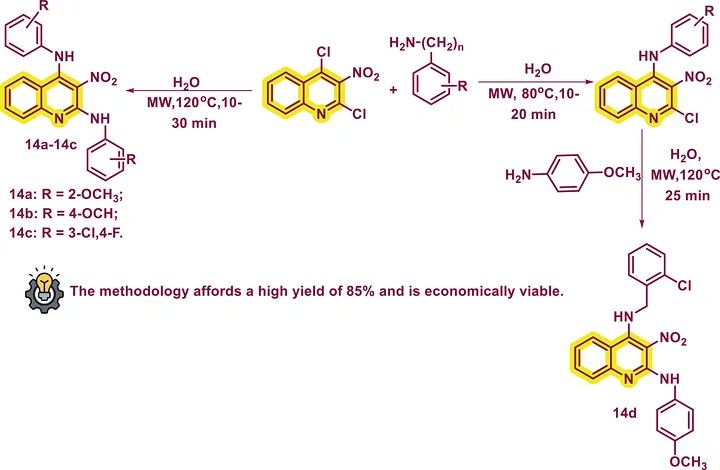
Microwave‐assisted synthesis of 4‐aryl‐substituted 2,4‐diaryl (dialkyl)amino‐3‐nitroquinolines (**14a–14d**).

The novel quinoline congeners were developed and assessed by Chauhan and associates against anti‐tumor agents. Using MAS, regioselective derivatives of 4‐aryl‐substituted 2,4‐diaryl (dialkyl)amino‐3‐nitroquinolines **(14a–14d)** (Scheme 13) were produced. The compounds' anticancer properties were measured using the MTT test. While compounds **14c** and **14d** showed significant activity against the colon cancer cell line HCT‐116‐p‐53 with IC_50_ values of 5.1 and 4.8 µM, respectively, compounds **14a** and **14b** demonstrated strong activity against the lung cancer cell lines A549 (IC_50_: 6.5 µM) and H‐460 (IC_50_: 5.4 µM) [92].

#### 2.1.1 Critical Analysis of MAS: Reaction Efficiency, Biological Activity, and SARs

**FIGURE 7:**
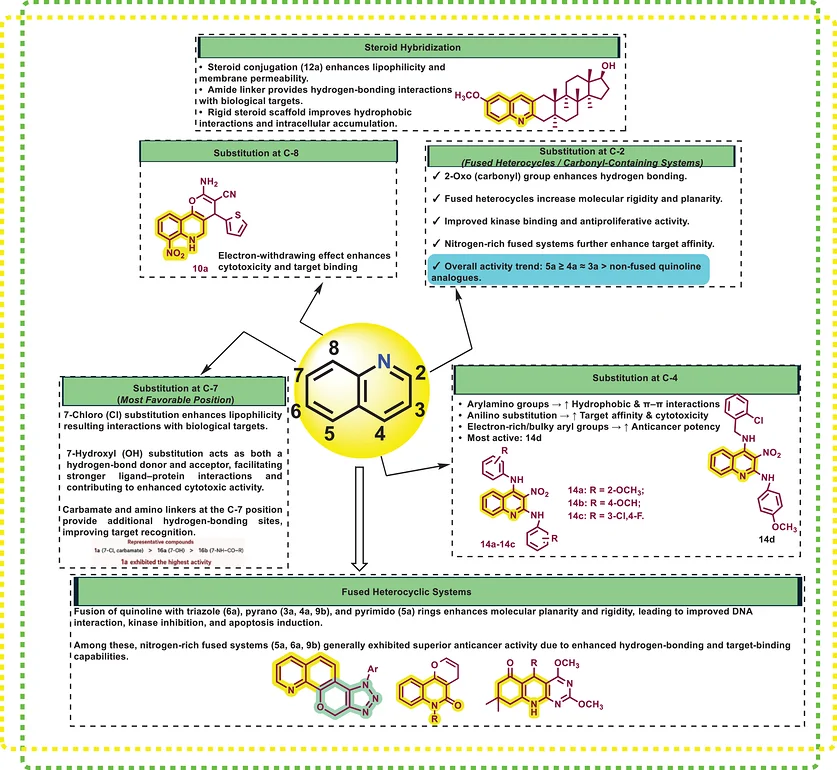
SAR summary of quinoline derivatives synthesized by using microwave‐assisted synthesis, highlighting the key pharmacophoric features and structural modifications responsible for the enhanced kinase inhibitory and antiproliferative activity of the tetrahydroquinoline scaffold.

MAS has essentially modified the workflow of modern medicinal chemistry by overcoming the limitations of classical conductive heating methods. A summary of the evaluation of its reaction efficiency, biological impacts, and SAR is depicted in the Figure 7. The classical thermal technology is slow and depends on conductive energy transfer from an external heat source through a reaction vessel wall, resulting in long reaction times, temperature gradients, and potential thermal deprivation. MAS completely circumvents these demerits by directly coupling with ionic conduction and dipolar rotation, enabling rapid and uniform heating. This results in the drop of reaction windows from hours to minutes or seconds. The reaction reported by Patel et al., Humal and co‐workers, and Arasakumar and his colleges involved three/multicomponent condensations or one‐pot synthesis and showed high yields ranging from 80% to 95%, while suggestively diminishing the formation of side products. Furthermore, these reactions were carried out under milder, ecofriendly solvent systems or solvent‐free conditions.

The effective parameters of MAS directly translate into cleaner compound libraries, which directly improves biological evaluation accuracy. The critical analysis of the reported literature showed microwave‐synthesized quinoline derivatives are strongly influenced by the nature and position of substituents on the quinoline scaffold. The SAR indicates that electron‐withdrawing groups (Cl and NO_2_), fused heterocyclic frameworks, arylamino substitution, and hydrogen‐bonding linkers are the key structural features responsible for enhanced anticancer activity. Substitution at C‐7 was most favorable, as the 7‐chloro carbamate derivative **1a** exhibited the highest potency, responsible for improved lipophilicity, membrane permeability, and hydrogen‐bonding interactions. Similarly, the 7‐hydroxy derivative **16a** showed amended activity, may be due to the facilitation of hydrogen‐bond formation with biological targets. Further, at the C‐2 position, fused heterocyclic systems, such as pyranoquinolines **(3a, 4a)** and pyrimidoquinoline **(5a)**, result in increased molecular rigidity and planarity, leading to enhanced kinase binding and antiproliferative activity. While, in comparison to other counterparts of quinoline derivatives, compounds **14d** at C‐4 arylamino substitution notably increased hydrophobic and *π*–*π* interactions, resulting in enhanced cytotoxicity. Electron‐withdrawing substituents further improved anticancer efficacy. The 8‐nitroquinoline derivative **(10a)** proved improved cytotoxicity, indicating that nitro groups favor target binding via electronic modulation. In addition, fused heterocyclic hybrids, including quinoline–triazole **(6a)** and triazolo‐pyranoquinoline **(9b)**, displayed amended anticancer activity due to increased structural rigidity and stronger interactions with biological targets. The steroid–quinoline hybrid **12a** also displayed promising activity, likely due to improved membrane permeability and intracellular accumulation. While comparing all derivatives, compound **1a** unrevealed as the most potent derivative because of the coactive effect of its 7‐chloro substituent and carbamate linker.

### 2.2 Synthesis of Quinoline Scaffold as Anticancer Agents Using UAS

**SCHEME 14:**
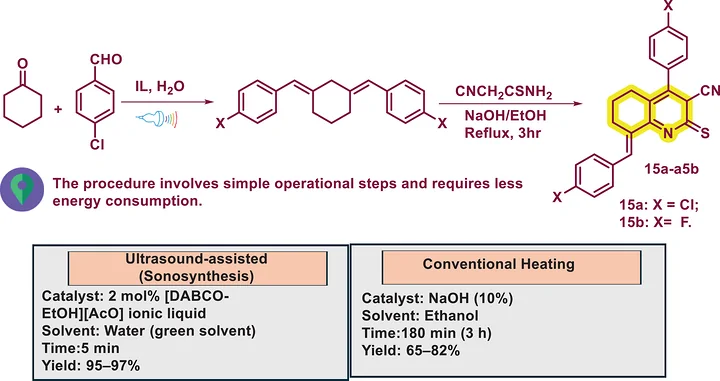
Sonochemistry‐assisted synthesis of tetrahydroquinoline analogues (**15a–15b**).

Sonochemistry exploits ultrasonic irradiation with a frequency of 20–100 kHz to produce improved regioselectivity [93], low energy consumption, modest operation, ease of use, speed up reaction rate, high percentage yield [94], and minimize waste [95]. Instead of heating the reaction to a high temperature, as is customary, the energy source in the ultrasound‐assisted reaction is ultrasonic irradiation [96]. Several medicinal chemists created the quinoline hybrids using sonochemistry. Owing to the advantages of Sonochemistry, tetrahydroquinoline analogues were synthesized and examined by Alanazi et al., as potential anti‐proliferative agents. Sono‐chemical synthesis of tetrahydroquinoline derivatives (**15a–15b**) (Scheme 14) was carried out with an ionic liquid catalyst. The produced sets were tested against HepG‐2, MCF‐7, and A549 cell lines, and doxorubicin was used as reference drug; the sets showed excellent antiproliferative activity, with IC_50_ values of 0.75, 1.02, and 1.27 µg/mL, respectively. Though among the developed series none of showed derivatives surpassed the reference drug. Compounds **15a** and **15b** were promising potential derivatives against HepG‐2 (IC_50_ = 2.86 and 11.23 µM) and MCF‐7 (IC_50_ = 3.17 and 13.14 µM) cell lines. With corresponding IC_50_ values of 25.54 and 28.45 µM, the compounds (**15a** and **15b**) demonstrated a modest level of efficacy against lung cancer (A549) cell lines [97].

**SCHEME 15:**
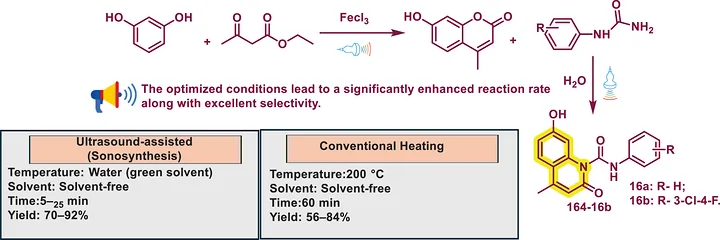
UAS of N‐phenyl‐7‐hydroxy‐4‐methyl‐2‐ oxoquinoline‐1(2*H*) carboxamide derivatives.

The designing strategy, environmentally friendly synthesis, and anticancer properties of novel quinoline series were discussed by Ali and associates. A panel of cancer cell lines was used to test the *N*‐Aryl‐7‐hydroxy‐4‐methyl‐2‐oxoquinoline‐1(2*H*) carboxamides (**16a–16b**) (Scheme 15). Compound **16a** showed strong action against TK‐10, UO‐31, HOP‐92, CAKI 1, and HS 578T (GI % = 82.90, 23.10, 16.00, 12.39, and 11.59 %). Similarly, compound **16b** had good efficacy against HOP‐92, A498, HOP‐62, UO‐31, CCRF‐CEM, with GI % of 58.61, 30.00, 27.44, 20.53, and 17.56, respectively. Imatinib, used as the reference drug, presented variable anti‐cancer activity across the NCI‐60 panel, with growth inhibition values ranging from 22.3% to 47.1% in representative cell lines. Even though imatinib exhibited wide antiproliferative activity, compound **16a** showed notably higher inhibition against the TK‐10 renal cancer cell line (82.90% GI), prominence the potential of this quinoline scaffold as anticancer agents [98].

**SCHEME 16:**
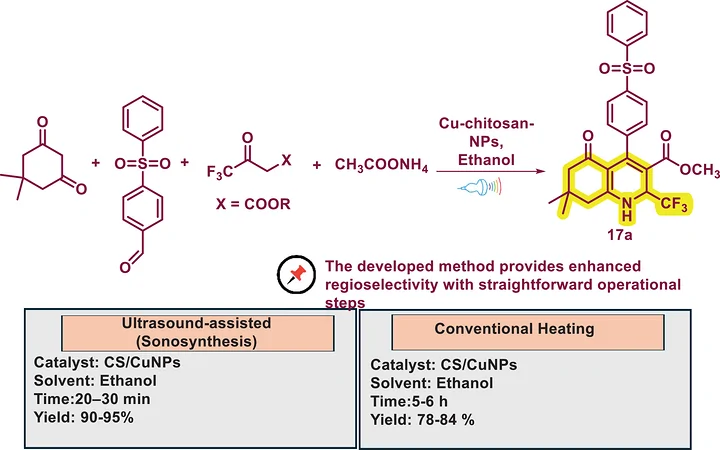
Chitosan‐mediated copper nanoparticles catalyze the ultrasonication‐based synthesis of novel tetrahydroquinolin‐5(1H)‐one derivatives (**17a**).

The quinoline compounds were synthesized using an ecofriendly route and tested for anticancer activity by Mokhtar et al. Chitosan‐mediated copper nanoparticles, which catalyze the ultrasonication‐based process, were employed for the synthesis of 4,6,7,8‐tetrahydroquinoline‐5(1H)‐ones (**17a**) (Scheme 16). The MCF‐7 cell line was used to test the produced compounds, and **17a** was the most potent among all, with an IC_50_ value of 0.002 µM as compared with staurosporine (IC_50_: 0.005 µM). Additionally, **17a** was tested for enzyme inhibition activity against EGFR, HER‐2, PDGFR, and VEGFR‐2 enzymes. Compound **17a** was found to be the most effective against EGFR, HER‐2, VEGFR, and PDGFR, with respective IC_50_ values of 0.11, 0.30, 0.21, and 1.05 µM. While, reference inhibitor sorafenib, demonstrated strong inhibition against all four kinases, with IC_50_ values of 0.04 for EGFR, 0.28 × 10^−3^ for HER‐2, 0.13 × 10^−3^ for PDGFR‐β, and 0.17 ± 0.02 µM for VEGFR‐2. Furthermore, compound **17a** also demonstrated cell arrest at the G2/M phase in cell cycle studies [99].

**SCHEME 17:**
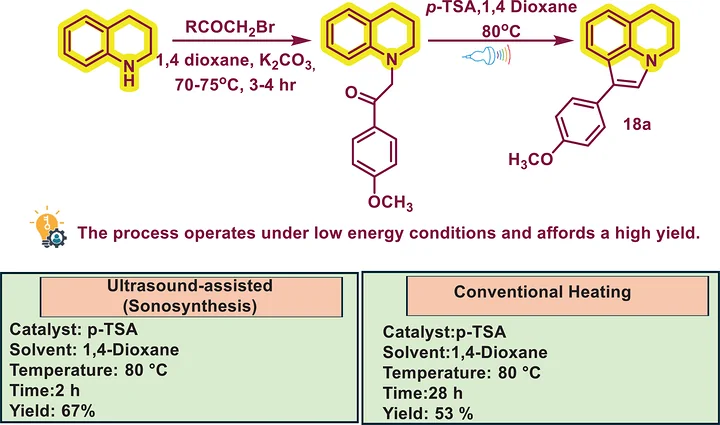
Ultrasonic‐bath‐mediated synthesis of pyrrole‐fused quinoline derivatives (**18a**).

The environmentally friendly green synthesis of substituted quinolines (Scheme 17) as anticancer agents was described by Rao and colleagues. Using an ultrasonic bath, pyrrole‐fused quinoline derivatives were created. The K562 cell lines were used to examine the anticancer properties of compound **18a**, which showed good effectiveness against the K562 cell lines by inhibiting growth (GI = 66%). Doxorubicin was used as the standard, demonstrating 82% inhibition at 10 µM, with the inhibitory effect gradually decreasing to 32% at 0.01 µM. While, compared with doxorubicin, compound **18a** exhibited modest antiproliferative activity, with 66%, 51%, 37%, and 23% inhibition at 10, 5, 1, and 0.1 µM, respectively, while no quantifiable activity was noticed at 0.01 µM [100].

#### 2.2.1 Critical Analysis of UAS: Reaction Efficiency, Biological Activity, and SARs

UAS notably enhanced the reaction productivity for the synthesis of quinoline derivatives by reducing reaction time and providing good‐to‐excellent yields. The tetrahydroquinoline derivatives **(15a–15b)** were synthesized in high yields under ultrasonic irradiation using an ionic liquid catalyst and unveiled promising antiproliferative activity against HepG‐2, MCF‐7, and A549 cell lines. Also, N‐aryl‐7‐hydroxy‐4‐methyl‐2‐oxoquinoline derivatives **(16a–16b)** were obtained in good yields and established broad‐spectrum anticancer activity, with **16a** showing superior growth inhibition against TK‐10 renal cancer cells. The UAS of tetrahydroquinolin‐5(1H)‐one derivative **(17a)** using chitosan‐supported Cu nanoparticles afforded an excellent yield and revealing remarkable cytotoxicity against MCF‐7 cells together with strong inhibition of EGFR, HER‐2, VEGFR‐2, and PDGFR. In contrast, the pyrrole‐fused quinoline derivative **(18a)** was synthesized efficiently under ultrasonic settings but exhibited only modest antiproliferative activity, indicating that high synthetic efficiency does not necessarily translate into superior biological potency.

**FIGURE 8:**
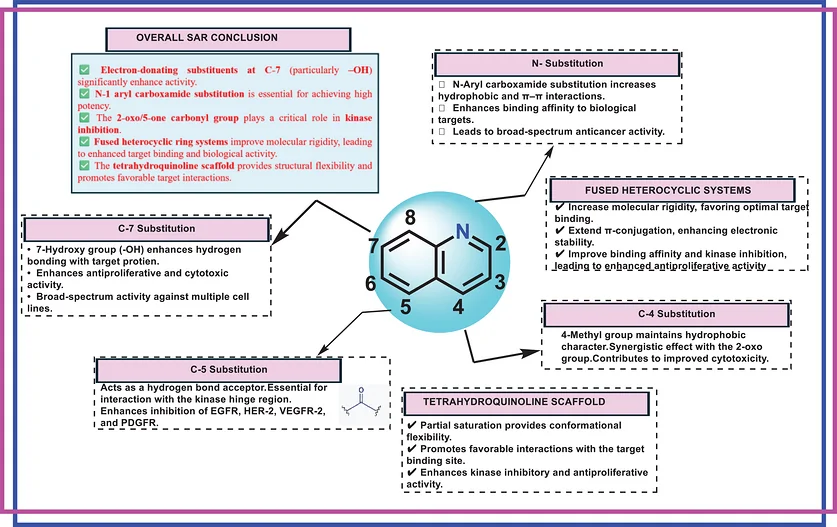
SAR summary of quinoline derivatives synthesized by using UAS, highlighting the key pharmacophoric features and structural modifications responsible for the enhanced kinase inhibitory and antiproliferative activity of the tetrahydroquinoline scaffold.

The SAR indicates (Figure 8) that the 7‐hydroxyl group improves hydrogen‐bonding interactions and anticancer activity, while the 2‐oxo and 5‐one carbonyl functionalities are crucial for kinase binding. N‐1 aryl carboxamide substitution expands hydrophobic and π–π interactions, whereas the 4‐methyl group donates to hydrophobic binding. Furthermore, the tetrahydroquinoline scaffold improves conformational flexibility, and pyrrole fusion intensify the molecular rigidity and π‐conjugation. The results demonstrated that biological activity was administered primarily by the nature and position of substituents on the quinoline scaffold rather than by the ultrasound‐assisted synthetic method itself.

## 3 Conclusion

Quinoline derivatives continue to occupy a central position in anticancer drug discovery due to their versatile chemical framework and ability to target multiple oncogenic pathways. Despite their therapeutic promise, traditional synthetic approaches for quinoline scaffolds are often associated with environmental and practical drawbacks, including harsh reaction conditions, excessive solvent usage, and poor sustainability. The adoption of green chemistry principles, particularly through microwave‐ and ultrasound‐assisted synthetic methodologies, has significantly transformed the synthesis of quinoline‐based anticancer agents.

As highlighted in this review, MAS offers rapid and uniform heating, reduced reaction times, and improved product yields, while UAS exploits acoustic cavitation to enhance reaction kinetics under mild conditions. Both approaches enable cleaner reactions, better atom economy, and reduced environmental impact, aligning well with the principles of sustainable chemistry. Importantly, numerous quinoline derivatives synthesized using these techniques have demonstrated promising anticancer activities across a wide range of cancer cell lines, along with notable enzyme inhibition and apoptosis‐inducing properties. The summary of the reported literature comparing compounds, yields, reaction conditions, and anticancer activities is tabulated in Table 2.

**TABLE 2:** Summary of green synthetic methodologies, reaction conditions, yields, and anticancer activities of quinoline derivatives.

Green method	Representative compound(s)	Optimized reaction conditions	Yield (%)	Reaction time	Most active derivative	Anticancer activity
Microwave	**1a–1b**	Solvent‐free, microwave irradiation	80–95	5–15 min	**1a**	IC_50_ = 0.16 µM (HCT‐116); potent HDAC inhibition
Microwave	**3a–4a**	Microwave‐assisted one‐pot synthesis	82–91	8–20 min	**3a**, **4a**	Potent antiproliferative activity against human cancer cell lines
Microwave	**5a**	Microwave‐assisted multicomponent synthesis	84–92	10–20 min	**5a**	Significant cytotoxic activity against cancer cells
Microwave	**10a–11b**	Microwave irradiation	70–91	2–20 min	**10a**	Improved cytotoxic activity due to nitro substitution
Microwave	**12a**	Microwave‐assisted synthesis of steroid–quinoline hybrid	High yield	Few minutes	**12a**	C33A (IC_50_ = 10.2 µM), MCF‐7 (19.1 µM), T47D (10.2 µM); induced apoptosis and G_0_/G_1_ arrest
Microwave	**13a–13b**	Green microwave synthesis	Good–excellent	Short	**13b**, **13a**	LOX IMVI (IC_50_ = 10.7 µM); EKVX (IC_50_ = 14.2 µM)
Microwave	**14a–14d**	Microwave‐assisted regioselective synthesis	Good–excellent	Minutes	**14d**, **14c**	HCT‐116 (IC_50_ = 4.8–5.1 µM); A549 (6.5 µM); H‐460 (5.4 µM)
Ultrasound	**15a–15b**	Ionic liquid catalyst, ultrasound irradiation	High	5 min	**15a**	HepG‐2 (IC_50_ = 2.86 µM), MCF‐7 (3.17 µM), moderate A549 activity
Ultrasound	**16a–16b**	Water, ultrasound irradiation	70–92	5–25 min	**16a**	TK‐10 (GI = 82.9%), active against UO‐31, HOP‐92, CAKI‐1 and HS578T
Ultrasound	**17a**	CS/CuNP catalyst, ultrasound	90–95	20–30 min	**17a**	MCF‐7 (IC_50_ = 0.002 µM); EGFR, HER‐2, VEGFR‐2 and PDGFR inhibition; G_2_/M arrest
Ultrasound	**18a**	Ultrasonic bath	Good	2 h	**18a**	K562 growth inhibition = 66% at 10 µM

Overall, microwave‐ and ultrasound‐assisted methodologies epitomize powerful and environmentally benign tools for the efficient synthesis of biologically active quinoline derivatives. Their successful application in anticancer research not only underscores their synthetic advantages but also highlights their potential to accelerate the discovery of next‐generation anticancer agents. Future efforts integrating these green techniques with rational drug design, mechanistic studies, and translational research are expected to further enhance the development of safe, effective, and sustainable quinoline‐based therapeutics. Furthermore, future research should emphasize on evolving sustainable, scalable, and industrially feasible synthetic processes by integrating continuous‐flow chemistry, recyclable heterogeneous catalysts, renewable feedstocks, solvent‐free or aqueous reaction systems, and energy‐efficient activation techniques. Major attention should give on catalyst reusability, solvent recycling, and complete green metric analysis to facilitate the translation of laboratory protocols into profitable manufacturing. From an industrial perspective, these developments have the potential to decrease production costs, energy consumption, and environmental impact while supporting regulatory necessities for sustainable pharmaceutical manufacturing.

In addition, the amalgamation of AI and ML with green chemistry offers exhilarating opportunities for fast‐tracking the discovery and optimization of quinoline‐based compounds. AI‐assisted drug design, reaction prediction, retrosynthetic planning, catalyst selection, and reaction‐condition optimization can meaningfully shorten development timelines while refining productivity and sustainability. The combination of computational tactics with environmentally benign synthetic strategies is anticipated to play a crucial role in the next generation of quinoline‐based drug discovery and pharmaceutical process development.

## Author Contributions

**Rohit Pal**: conceptualization, literature search, data curation, formal analysis, visualization, writing – original draft preparation, and manuscript editing. **Gurubasavaraja Swamy Purawarga Matada**: conceptualization, methodology, supervision, validation, critical review, manuscript editing, and final approval. **Ghanshyam Teli**: literature review, data interpretation, writing – review and editing, and critical revision of the manuscript. **Manjushree B. V**.: literature review, data curation, visualization, writing – review and editing. **Vipin Kumar Pandey**: data interpretation, critical review, writing – review and editing. **Shourya Pratap**: literature review, validation, writing – review and editing. All authors contributed to the preparation, revision, and approval of the final manuscript. All authors have read and agreed to the published version of the manuscript.

## Ethics Statement

This article is based exclusively on previously published literature and does not involve any studies with human participants or animals performed by any of the authors. Therefore, ethical approval was not required.

## Conflicts of Interest

The authors declare no conflicts of interest.

## Use of Generative AI and AI‐Assisted Technologies in the Writing Process

The authors used AI‐assisted tools solely to improve the language, grammar, and readability of the manuscript. ChatGPT, OpenAI was also used to prepare the graphical illustration (Figure 4). The scientific content and accuracy were verified by the authors. The scientific content, literature selection, critical analysis, interpretation, and conclusions were developed independently by the authors, who assume full responsibility for the integrity and accuracy of the work.

## Data Availability

No new data were generated or analyzed during the preparation of this review article. All data discussed in this manuscript were obtained from previously published studies, which have been appropriately cited. Therefore, data sharing is not applicable to this article.
